# Exploring histone loading on HIV DNA reveals a dynamic nucleosome positioning between unintegrated and integrated viral genome

**DOI:** 10.1073/pnas.1913754117

**Published:** 2020-03-11

**Authors:** Shinichi Machida, David Depierre, Heng-Chang Chen, Suzie Thenin-Houssier, Gaël Petitjean, Cecile M. Doyen, Motoki Takaku, Olivier Cuvier, Monsef Benkirane

**Affiliations:** ^a^Institut de Génétique Humaine, Université de Montpellier, Laboratoire de Virologie Moléculaire CNRS-UMR9002, 34000 Montpellier, France;; ^b^Laboratoire de Biologie Moléculaire Eucaryote, Centre de Biologie Intégrative, Université de Toulouse, CNRS-UMR5099, 31000 Toulouse, France;; ^c^Department of Biomedical Sciences, University of North Dakota School of Medicine, Grand Forks, ND 58202

**Keywords:** HIV, unintegrated viral DNA, histone, nucleosome positioning, transcription

## Abstract

The aim of the present study was to understand the biology of unintegrated HIV-1 DNA and reveal the mechanisms involved in its transcriptional silencing. We found that histones are loaded on HIV-1 DNA after its nuclear import and before its integration in the host genome. Nucleosome positioning analysis along the unintegrated and integrated viral genomes revealed major differences in nucleosome density and position. Indeed, in addition to the well-known nucleosomes Nuc0, Nuc1, and Nuc2 loaded on integrated HIV-1 DNA, we also found NucDHS, a nucleosome that covers the DNase hypersensitive site, in unintegrated viral DNA. In addition, unintegrated viral DNA-associated Nuc0 and Nuc2 were positioned slightly more to the 5′ end relative to their position in integrated DNA. The presence of NucDHS in the proximal region of the long terminal repeat (LTR) promoter was associated with the absence of RNAPII and of the active histone marks H3K4me3 and H3ac at the LTR. Conversely, analysis of integrated HIV-1 DNA showed a loss of NucDHS, loading of RNAPII, and enrichment in active histone marks within the LTR. We propose that unintegrated HIV-1 DNA adopts a repressive chromatin structure that competes with the transcription machinery, leading to its silencing.

## Significance

The biology of HIV DNA, from its synthesis to its integration into the host genome, remains poorly understood. Here we show that in the nucleus, histones are rapidly loaded on newly synthesized unintegrated HIV DNA. Interestingly, the chromatin architecture around the HIV long terminal repeat (LTR) is different in unintegrated and integrated HIV DNA. Specifically, a nucleosome present only on the DNase hypersensitive site of unintegrated HIV DNA contributes to the transcriptional silencing of unintegrated HIV DNA by preventing RNAPII recruitment.

Early in the life cycle of HIV, on fusion of the viral and cellular membranes and release of the viral core in the host cell cytoplasm, the viral RNA genome is reverse-transcribed into a double-stranded linear viral DNA (dslvDNA). This dslvDNA, associated with the viral integrase (IN) and various viral and cellular proteins, forms a large nucleoprotein complex, the preintegration complex (PIC) (1, 2). Lentiviruses, including HIV-1, can infect nondividing cells, and they enter the nucleus through an active process involving the nuclear pore complex (NPC) (1, 2). Although the exact PIC composition remains controversial and poorly described (3), PIC components are needed for the successful nuclear import and stable integration of dslvDNA in the host genome. Once in the nucleus, HIV integration occurs in the outer shell of the nucleus close to the nuclear pore, preferentially into transcriptionally active chromatin regions (4–9).

Nuclear import and integration are tightly linked and influence dslvDNA integration efficiency and localization, two features essential for productive infection and viral persistence in infected cells (8, 9). These two steps of the viral cycle are poorly understood, but it is known that HIV hijacks the cellular machinery, and that viral and cellular proteins are both essential for nuclear import and integration (2, 3). Precisely determining HIV DNA biology from its synthesis to integration represents a step toward a better understanding of the mechanisms regulating integration site selection and viral transcription.

Nucleosomes, the basic structural units of chromatin, package and regulate eukaryotic genomes (10–12). Nucleosomes are composed of approximately 146 bp of DNA wrapped around a histone octamer consisting of two copies of each core histone (H2A, H2B, H3, and H4) (13). DNA methylation, histone chaperons, nucleosome remodeling complexes, and histone modifiers play major roles in chromatin dynamics by establishing the epigenetic state that modulates DNA accessibility and consequently regulates genome functions, such as DNA replication, repair, and transcription (10–12). It has been shown that nucleosome sliding and disassembly accompany gene activation by enhancing promoter accessibility to the transcription machinery (10, 12, 14). On integration in the host genome, HIV-1 DNA adopts a chromatin structure with precisely positioned nucleosomes (Nuc0, Nuc1, and Nuc2) and a DNase hypersensitive site (DHS) around the HIV long terminal repeat (LTR) (15, 16).

It is well established that HIV-1 gene expression is regulated through two rate-limiting steps: chromatin remodeling and RNA polymerase II (RNAPII) pausing induced by the recruitment of negative elongation factor (NELF) (15–19). Activation of HIV-1 gene expression is accompanied by chromatin remodeling, particularly at the repressive Nuc1 positioned immediately downstream of the transcription start site (TSS) (15, 16); however, it is unclear when, where, and how histones are loaded on reverse-transcribed HIV DNA. Goff’s group reported that histones are loaded on Moloney murine leukemia virus (MLV) vDNA after nuclear entry and before integration, and that histone modifications of chromatinized MLV linear and circular DNA are detected after histone loading (20). More recently, they identified the nuclear double-stranded DNA-binding protein NP220 as a transcriptional repressor of unintegrated MLV DNA (21). NP220 recruits the HUSH complex (consisting of MPP8, TASOR, and PPHLN1) and the histone methyltransferase SETDB1 at the LTR of MLV and silences unintegrated MLV DNA (21). These findings strongly suggest that the unintegrated MLV promoter is actively repressed. However, knockout of HUSH complex components and of SETDB1 had no/minimal effect on the silencing of unintegrated HIV-1 DNA (21). Thus, it is unclear how unintegrated HIV DNA is silenced. In this study, we show that unintegrated HIV DNA is loaded with histones and reveal unexpected chromatin architecture dynamics in unintegrated and integrated HIV DNA.

## Results

### Histones Are Loaded on Unintegrated HIV DNA.

**Fig. 1.**
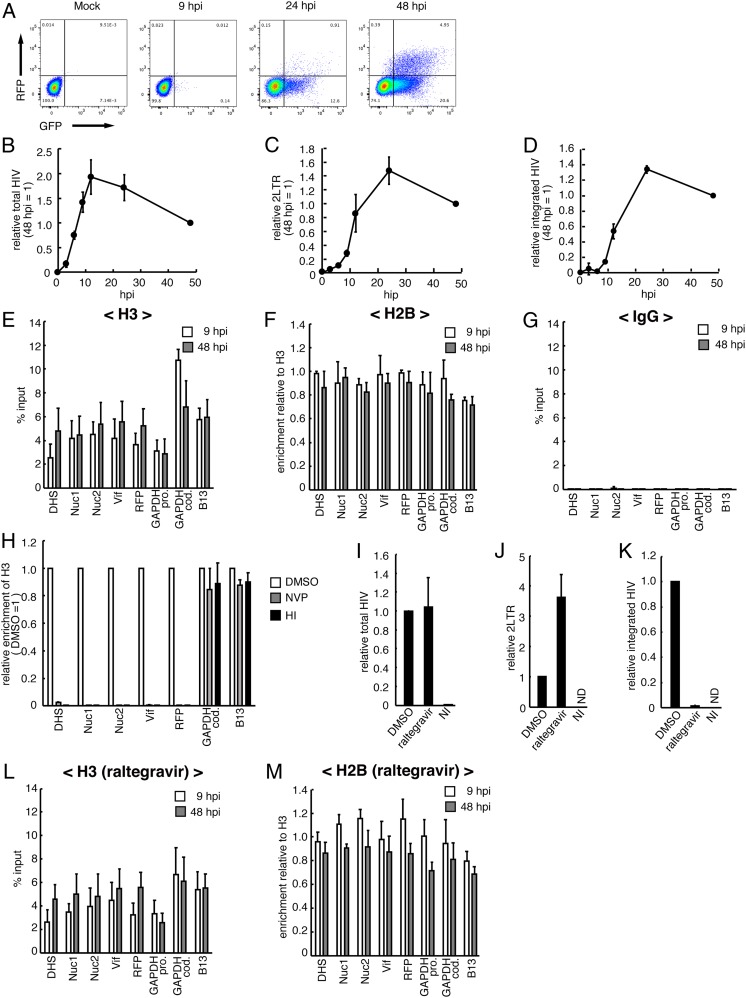
Histones rapidly associate with incoming HIV DNAs. (*A*) Flow cytometry analysis of RFP and GFP expression in Jurkat cells infected with VSV-G pseudotyped HIV-1 containing the two-color reporters at 9, 24, and 48 hpi. Representative images of one of three independent experiments are shown. (*B*–*D*) Quantification of total HIV DNA (*B*), 2LTR circles (*C*), and integrated HIV DNA (*D*) in Jurkat cells infected with VSV-G pseudotyped HIV-1 containing the two-color reporters. Data are represented as quantification relative to the value at 48 hpi. The mean values ± SD of three independent experiments are plotted. (*E*–*G*) ChIP assays performed at 9 and 48 hpi using anti-H3 (*E*), anti-H2B (*F*), and nonspecific IgG (*G*) antibodies, followed by qPCR analysis with specific primers for HIV (DHS, Nuc1, Nuc2, Vif, and RFP), the *GAPDH* promoter region, the *GAPDH* coding region, and the B13 genomic region. The mean ± SD values of three independent experiments are plotted. (*H*) ChIP assays using Jurkat cells infected with VSV-G pseudotyped HIV-1 in the absence (DMSO) or presence of 1 µM nevirapin (NVP), or infected with heat-inactivated (HI) viruses. ChIP assays were performed at 24 hpi using an anti-H3 antibody, followed by qPCR analysis with the primers listed in *E*–*G*. The mean ± SD values of three independent experiments are plotted. (*I*–*K*) Quantification of total HIV DNA (*I*), 2LTR circles (*J*), and integrated HIV DNA (*K*) in Jurkat cells infected with VSV-G pseudotyped HIV-1 containing the two-color reporters in the absence (DMSO) or presence of raltegravir (1 µM). DNA was extracted from infected cells at 24 hpi. Data are presented as the quantification relative to control (incubation with DMSO). NI, noninfected cells; ND, not detected. The mean ± SD values of three independent experiments are plotted. (*L* and *M*) ChIP assays using Jurkat cells infected with HIV-1 containing the two-color reporters in the presence of raltegravir (1 µM). ChIP assays were performed at 9 and 48 hpi using anti-H3 (*L*) and anti-H2B (*M*) antibodies, followed by qPCR analysis with the primers listed in *E*–*G*. The mean ± SD values of three independent experiments are plotted.

To investigate histone loading on HIV DNA, we infected Jurkat cells with VSV-G pseudotyped HIV-1 engineered to express RFP under the control of the LTR (rfp fused to the 3′ end of nef) and GFP under the control of the CMV promoter (CMV-gfp transcriptional unit inserted after nef-rfp). FACS analysis at 9, 24, and 48 h postinfection (hpi) showed that RFP and GFP expression could be detected starting at 24 hpi (Fig. 1*A*). We next analyzed the kinetics of total HIV DNA (Fig. 1*B*), 2LTR circles (Fig. 1*C*), and viral integration (Fig. 1*D*). We found that at 9 hpi, the linear HIV DNA form was predominant compared with 2LTR circles and integrated forms. Conversely, at 48 hpi, the integrated forms were predominant, and the 2LTR circles represented less than 20% of the total HIV DNA. We then performed chromatin immunoprecipitation (ChIP) experiments using chromatin prepared from infected Jurkat cells at 9 and 48 hpi and anti-histone H3 and H2B antibodies or nonspecific IgG (as a control) (Fig. 1 *E*–*G*). We assessed the presence of HIV DNA and control genomic loci in immunoprecipitated chromatin by qPCR using specific primers. Histones H3 and H2B were loaded along HIV DNA at 9 hpi, with no significant increase at 48 hpi (Fig. 1 *E*–*G*). Moreover, H3 and H2B were loaded on newly reverse-transcribed viral DNA, because incubation with nevirapin and infection with heat-inactivated HIV-1 resulted in loss of the H3 signal at HIV DNA, with no effect on the ChIP signals at genomic loci (Fig. 1*H*).

These findings indicate that histones H3 and H2B are loaded on newly synthesized unintegrated HIV DNA (uniHIV DNA). To strengthen this conclusion, we performed a similar experiment using the integrase inhibitor raltegravir. As expected, incubation with raltegravir had no effect on HIV DNA synthesis (Fig. 1*I*), increased HIV DNA 2LTR circle forms (Fig. 1*J*), and strongly reduced HIV DNA integration in the host genome compared with cells incubated with dimethyl sulfoxide (DMSO) (Fig. 1*K*). However, raltegravir had no effect on H3 and H2B loading on HIV DNA at 9 and 48 hpi (Fig. 1 *L* and *M*). Thus, chromatinization of newly synthesized HIV DNA occurs before its integration.

### Nuclear Import of HIV DNA Is Required for Histone Deposition.

**Fig. 2.**
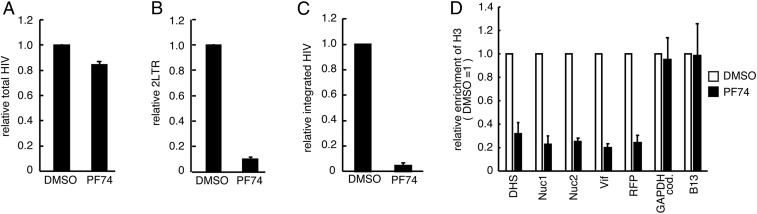
Histone loading on HIV DNA requires nuclear import of HIV DNA. (*A*–*C*) Quantification of total HIV DNA (*A*), 2LTR circles (*B*), and integrated HIV DNA (*C*). Differentiated THP-1 cells incubated with virus-like particles containing Vpx (VLP-Vpx) were infected with VSV-G pseudotyped HIV-1 containing the two-color reporters in the absence (DMSO) or presence of PF74 (2 µM). DNA was extracted from infected cells at 24 hpi. Data are presented as quantification relative to DMSO. (*D*) Differentiated THP-1 cells incubated with VLP-Vpx were infected with VSV-G pseudotyped HIV-1 in the absence or presence of PF74 (2 µM). ChIP assays were performed at 24 hpi using an anti-H3 antibody, followed by qPCR analysis with primers specific for HIV (DHS, Nuc1, Nuc2, Vif, and RFP), the *GAPDH* coding region, and the B13 genomic region. The mean ± SD values of three independent experiments are plotted.

To determine whether nuclear import is required for HIV DNA chromatinization, we performed ChIP experiments in the presence of PF74, which prevents the binding of CPSF6 and Nup153 to HIV capsids and consequently inhibits the nuclear import of HIV DNA (22–26). For this experiment, we differentiated THP-1 monocytic cells by incubation with phorbol 12-myristate 13-acetate (PMA) to prevent the nuclear import of HIV DNA through the nuclear membrane breakdown that occurs during the cell cycle. To evaluate the PF74-dependent inhibition of HIV DNA nuclear import, we infected differentiated THP-1 cells, which were incubated with viral protein X (Vpx) to overcome SAMHD1-mediated viral restriction (27–29), with HIV-1 in the presence or absence of PF74 (Fig. 2 *A*–*C*). PF74 did not affect total HIV DNA accumulation (Fig. 2*A*) but drastically reduced 2LTR circles (Fig. 2*B*) and HIV integration (Fig. 2*C*) compared with DMSO-treated cells, indicating that PF74 efficiently blocks HIV DNA nuclear import in differentiated THP-1 cells. ChIP using an anti-H3 antibody showed a significant decrease in H3 loading on HIV DNA in PF74-treated differentiated THP-1 cells compared with DMSO-treated cells. Conversely, the ChIP signals at the human genomic loci were comparable in the two conditions (Fig. 2*D*). These findings demonstrate that histone loading on HIV DNA requires its nuclear import.

### Dynamic Nucleosome Positioning Along Unintegrated HIV DNA.

**Fig. 3.**
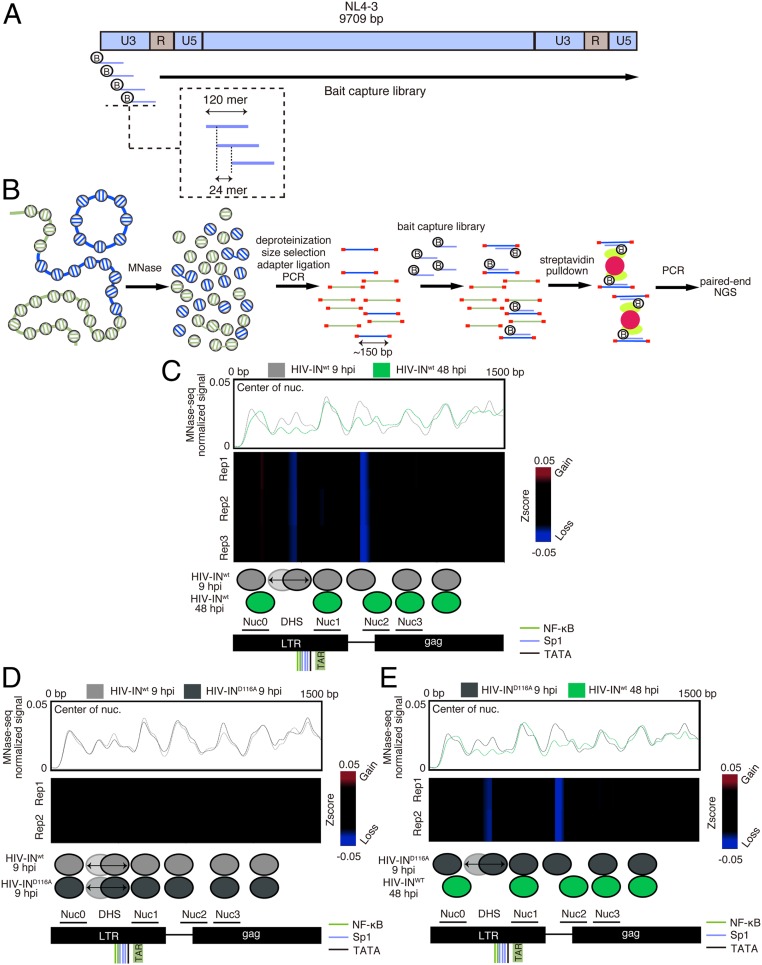
The local chromatin architecture around LTR is dynamically changed at the early infection phase. (*A*) Representation of biotinylated RNA baits that target HIV for capture MNase-seq. (*B*) Experimental strategy of the capture MNase-seq approach. Chromatin extracted from cells infected with VSV-G pseudotyped NL4-3-IN^WT^ or NL4.3-IN^D116A^ was digested with MNase. After deproteinization, mononucleosome fragments were extracted, and adapters were ligated. After PCR amplification, fragments derived from HIV were hybridized to the RNA capture library, followed by streptavidin pull-down. The resulting capture MNase libraries were analyzed by paired-end sequencing. (*C*) MNase-seq profiles along the first 1,500 bp of HIV DNA in HIV-IN^wt^ samples at 9 and 48 hpi. (*Top*) The coverage corresponding to the nucleosome center. (*Middle*) Differential heatmap showing the nucleosome coverage variations along the first 1,500 bp of HIV DNA. Blue indicates loss of signal; red, gain of signal. Differential heatmaps are filtered to show only the validated significantly different loci with a false discovery rate (FDR) <1e-3 (*SI Appendix*, *Materials and Methods*). (*Bottom*) Genome annotation with the nucleosome positioning corresponding to HIV-IN^wt^ at 9 and 48 hpi. Binding sites for NF-κB and SP1, TATA, and TAR are depicted in the genome annotation. Previously described nucleosome positions in the integrated vDNA (15) are also represented in the genome annotation. (*D*) Same as *C* for the comparison between HIV-IN^wt^ and HIV-IN^D116A^ at 9 hpi. (*E*) Same as *C* for the comparison between HIV-IN^D116A^ at 9 hpi and HIV-IN^wt^ at 48 hpi.

Our foregoing results revealed that histone proteins are loaded on uniHIV DNA in the nucleus before its integration. We next determined nucleosome positioning in unintegrated and integrated HIV DNA. For this purpose, we performed a capture-MNase-seq analysis using chromatin prepared from Jurkat cells infected with integrase wild-type (IN^wt^) or integrase mutant (IN^D116A^) HIV-NL4.3, using a low multiplicity of infection (MOI) of 0.2 to avoid overloading infected cells with viral DNA. Quantification of total HIV DNA, 2LTR circles, and integrated viral DNA by qPCR (*SI Appendix*, Fig. S1) showed that 2LTR circles represented 5% at 9 hpi and 12% at 48 hpi of the total HIV DNA for HIV-IN^wt^ and 26% at 9 hpi and 100% at 48 hpi of the total HIV DNA for HIV-IN^D116A^. As we used a low MOI, we first captured MNase-treated mononucleosome fragments using a custom-designed collection of biotinylated RNA baits targeting HIV (Fig. 3 *A* and *B* and *SI Appendix*, Fig. S2), and then analyzed them by deep sequencing.

Comparative analyses of nucleosome positioning along the IN^wt^ and IN^D116A^ HIV genomes at 9 and 48 hpi highlighted major changes in nucleosome density and positioning in the LTR region of IN^wt^ vDNA from 9 to 48 hpi (Fig. 3*C* and *SI Appendix*, Fig. S3). The even mapping of sonicated fragments to the HIV genome excluded a bias in the capture experiments (*SI Appendix*, Fig. S4). Specifically, the nucleosome map for integrated vDNA at 48 hpi was identical to that reported previously (15). Conversely, in univDNA, nucleosome density was increased, and nucleosome positioning at the LTR region was changed (Fig. 3*C*; compare 9 hpi and 48 hpi). Indeed, the positions of Nuc0 and Nuc2 in univDNA were slightly upstream of their known positions in integrated vDNA (Fig. 3*C*). We also identified a nucleosome positioned at the DHS (NucDHS) between Nuc0 and Nuc1 in univDNA but not in integrated DNA (Fig. 3*C*). In HIV-IN^D116A^ at 9 hpi, the nucleosome map was identical to that of uniHIV-IN^wt^ (9 hpi) (Fig. 3*D*). Moreover, we detected similar changes in nucleosome positioning in HIV-IN^D116A^ at 9 hpi and integrated HIV-IN^wt^ at 48 hpi (Fig. 3*E*). Taken together, these experiments show that nucleosomes are loaded on univDNA and highlight the nucleosome position dynamics particularly at the LTR region: disassembly of one nucleosome (NucDHS) and sliding of two nucleosomes (Nuc0 and Nuc2) on integration.

### RNAPII Loading and the Epigenome of Unintegrated and Integrated HIV DNA.

**Fig. 4.**
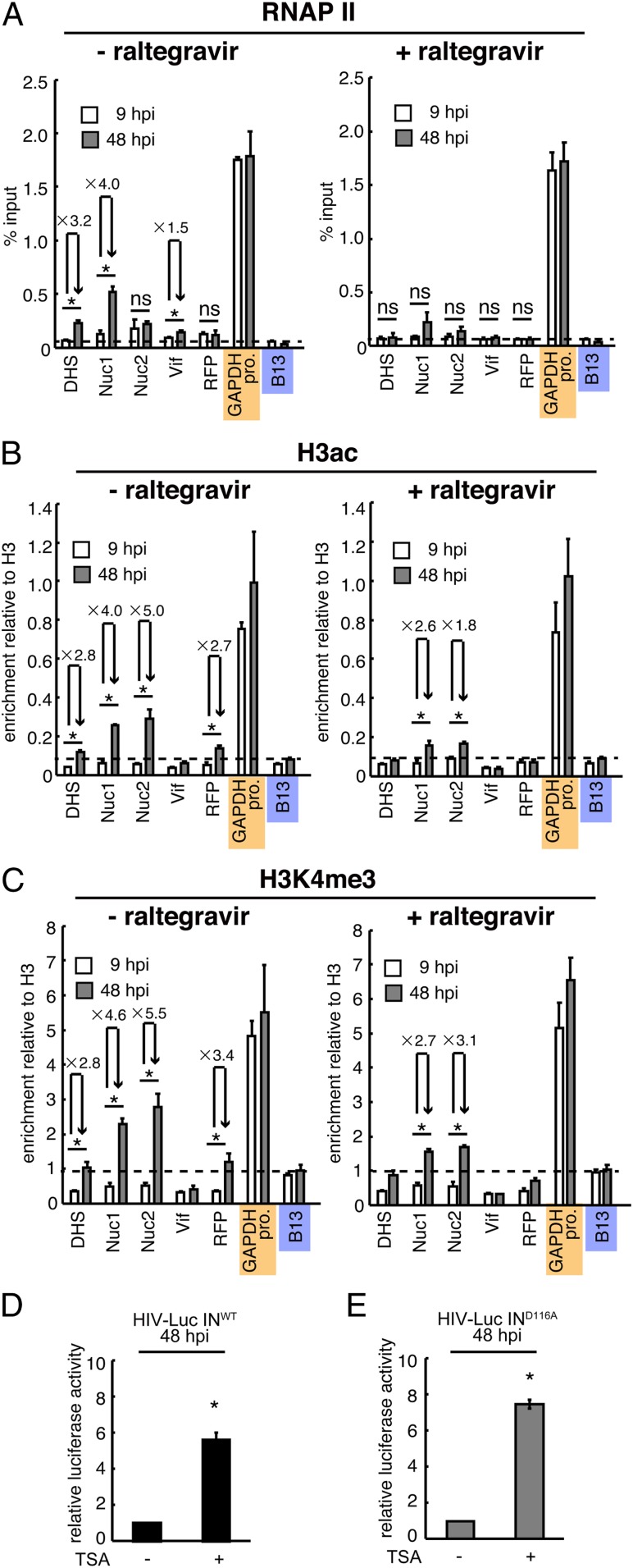
RNAPII and active histone marks are preferentially loaded on integrated HIV DNA. (*A*–*C*) ChIP assays using Jurkat cells infected with HIV-1 containing the two-color reporters. ChIP assays were performed at 9 and 48 hpi using anti-RNAPII (*A*), anti-H3ac (*B*), and anti-H3K4me3 (*C*) antibodies in the absence (*Left*) or presence (*Right*) of raltegravir (1 µM). The mean ± SD values of three independent experiments are plotted. *P < 0.05, independent Student’s *t* test. Orange and blue marks indicate positive and negative controls, respectively. (*D*) Luciferase assays were performed using Jurkat cells infected with VSV-G pseudotyped HIV-Luc IN^wt^ or HIV-Luc IN^D116A^ at 48 hpi in the absence or presence of 0.5 µM TSA. Data are presented as quantification relative to the value in the absence of TSA. The mean ± SD values of three independent experiments are plotted. *P < 0.05, independent Student’s *t* test.

MNase-seq data analysis revealed the dynamics of HIV DNA-associated nucleosomes (Fig. 3) and the presence of NucDHS only on unintegrated HIV DNA. The NucDHS position along the promoter-proximal region containing the TATA box, Sp1, and NF-κB–binding sites suggests that NucDHS may impose a considerable barrier to the recruitment of the transcription machinery, including RNAPII, thus preventing transcription from univDNA. To test this hypothesis, we performed ChIP experiments using an anti-RNAPII antibody and chromatin from HIV-1–infected Jurkat cells that were incubated with or without raltegravir and harvested at 9 and 48 hpi (Fig. 4*A*). We found that at 9 hpi, the level of RNAPII associated with vDNA was similar or slightly higher than that found at the B13 genomic region used as a negative control (Fig. 4*A*, *Left*). At 48 hpi, RNAPII recruitment at DHS and Nuc1 was significantly increased compared with that at 9 hpi (Fig. 4*A*, *Left*; compare 9 and 48 hpi). Raltegravir prevented RNAPII recruitment to HIV DNA at 48 hpi (Fig. 4*A*, *Right*) without affecting RNAPII loading on the *GAPDH* promoter (positive control).

To better define the mechanism underlying the absence of transcription from unintegrated HIV DNA, we determined the epigenetic marks associated with active transcription, particularly H3ac, which is associated with active chromatin, and H3K4me3, which marks active promoters (30–32). We confirmed the specificity of the anti-H3ac and anti-H3K4me3 antibodies for ChIP experiments using positive and negative controls corresponding to well-characterized genomic loci: *GAPDH* promoter enriched in H3ac and H3K4me3 and a B13 negative control region on chromosome 19. We found that the levels of H3ac and H3K4me3 associated with unintegrated HIV DNA were lower than in the negative control at 9 hpi but were increased at 48 hpi (Fig. 4 *B* and *C*, *Left*). This increase was limited in raltegravir-treated samples (Fig. 4 *B* and *C*; compare *Right* and *Left*), suggesting that these active histone marks are loaded after HIV DNA integration. Taken together, these experiments show that unintegrated HIV DNA adopts a chromatin structure that creates a barrier to RNAPII recruitment and that lacks active histone marks to prevent transcription.

To strengthen this conclusion, we analyzed the effect of the histone deacetylase inhibitor trichostatin A (TSA) on the activity of HIV-Luc-IN^D116A^ LTR. As TSA has been shown to enhance transcription from integrated viruses, HIV-Luc-IN^wt^ served as a positive control. TSA treatment of HIV-Luc-IN^D116A^–infected Jurkat cells resulted in enhanced luciferase activity (Fig. 4*E*), comparable to that observed for HIV-Luc-IN^wt^ (Fig. 4*D*). This result highlights the role of chromatin in regulating transcription from both unintegrated and integrated viral genomes.

**Fig. 5.**
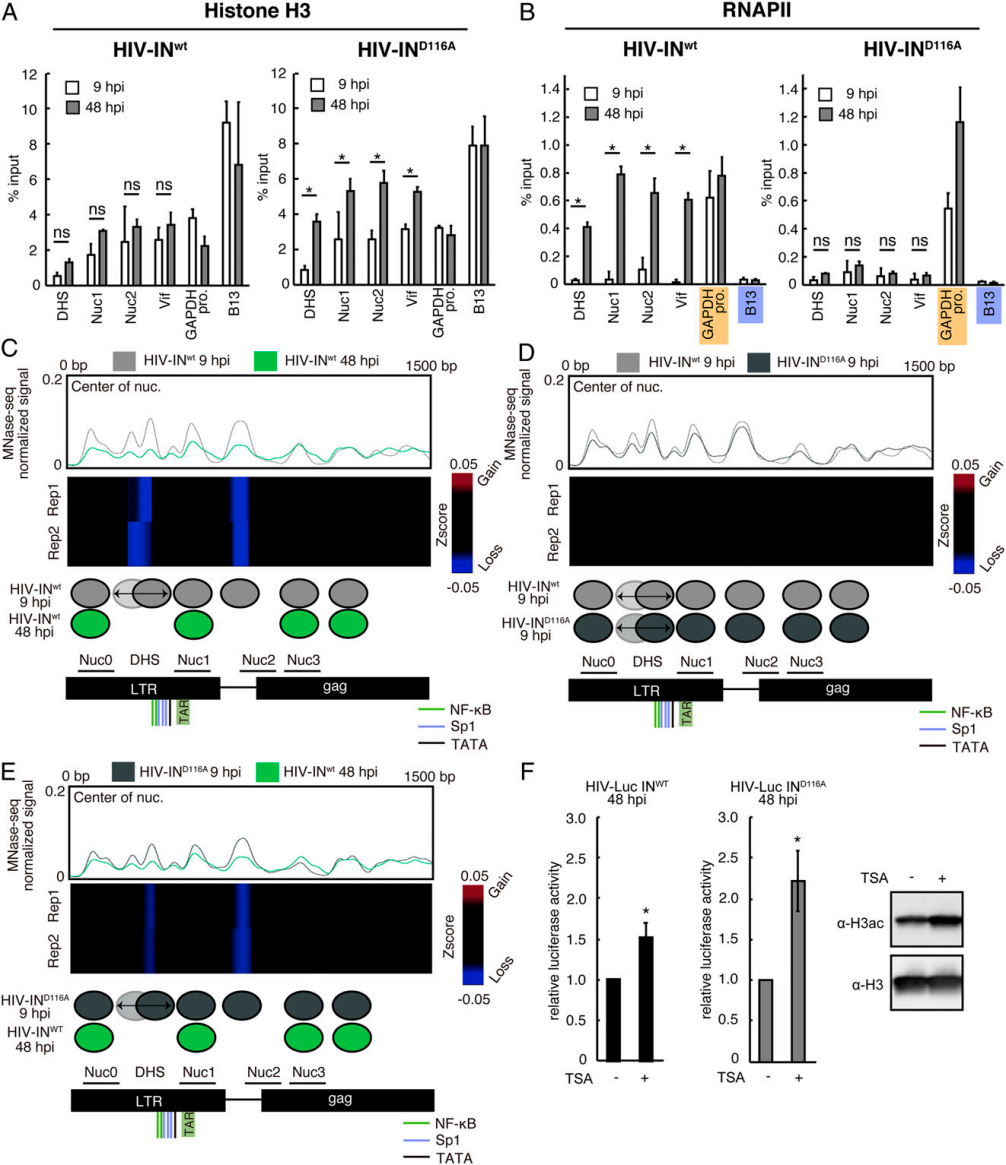
A nucleosome covering of the DHS prevents RNAPII loading on unintegrated HIV DNA in primary CD4 T cells. (*A* and *B*) ChIP assays using primary CD4 T cells infected with VSV-G pseudotyped NL4-3-IN^WT^ or NL4.3-IN^D116A^. ChIP assays were performed at 9 and 48 hpi using anti-histone H3 (*A*) and anti-RNAPII (*B*) antibodies. The mean ± SD values of three independent experiments are plotted. *P < 0.05, independent Student’s *t* test. Orange and blue marks indicate positive and negative controls, respectively. (*C*) MNase-seq profiles along the first 1,500 bp of HIV DNA for HIV-IN^wt^ at 9 and 48 hpi. (*Top*) The coverage corresponding to the nucleosome center. (*Middle*) Differential heatmap showing variations in nucleosome coverage along the first 1,500 bp of HIV DNA. Blue indicates loss of signal; red, gain of signal. Differential heatmaps are filtered to show only validated significantly different loci with an FDR <1e-3 (*SI Appendix*, *Materials and Methods*). (*Bottom*) Genome annotation with the nucleosome positioning corresponding to HIV-IN^wt^ at 9 and 48 hpi. Binding sites for NF-κB and SP1, TATA, and TAR are depicted in the genome annotation. Previously described nucleosome positions in the integrated vDNA (15) are also represented in the genome annotation. (*D*) Same as *C* for the comparison between HIV-IN^wt^ and HIV-IN^D116A^ at 9 hpi. (*E*) Same as *C* for the comparison between HIV-IN^D116A^ at 9 hpi and HIV-IN^wt^ at 48 hpi. (*F*) Luciferase assays were performed using primary CD4 T cells infected with VSV-G pseudotyped HIV-Luc IN^wt^ (*Left*) and IN^D116A^ (*Middle*) in the absence or presence of TSA (125 nM). Data are presented as the quantification relative to the value in the absence of TSA. The mean ± SD values of three independent experiments are plotted. *P < 0.05, independent Student’s *t* test. H3ac and H3 levels in primary CD4T cells treated without or with TSA (125 nM) were analyzed by Western blotting (*Right*).

We next used primary CD4 T cells to assess histone loading, RNAPII occupancy, and nucleosome positioning on unintegrated and integrated vDNA (Fig. 5). ChIP experiments using anti-H3 and anti-RNAPII antibodies and chromatin prepared from PHA/IL2-activated primary CD4 T cells infected with HIV-IN^wt^ or HIV-IN^D116A^ showed that at 9 hpi, histone H3 was loaded on HIV-IN^wt^ and HIV-IN^D116^ DNA, and that at 48 hpi, H3 loading was significantly increased on HIV-IN^D116^ DNA but not on HIV-IN^wt^ DNA (Fig. 5*A*). Quantification of HIV DNA synthesis showed that total HIV DNA was present at 9 hpi; 2LTR circle forms represented 2% of the total HIV DNA. The absence of integration events for HIV-IN^wt^ and HIV-IN^D116A^ DNA at this time point (*SI Appendix*, Fig. S5) suggests that H3 is loaded on linear HIV DNA before integration. Moreover, we found that at 9 hpi, RNAPII was undetectable in both HIV-IN^wt^ and HIV-IN^D116A^, but at 48 hpi, RNAPII was massively recruited along the HIV-1 genome in HIV-IN^wt^, but not HIV-IN^D116A^, samples (Fig. 5*B*).

We then performed MNase-seq to assess nucleosome positioning at 9 and 48 hpi (Fig. 5 *C*–*E* and *SI Appendix*, Figs. S6 and S7). As observed in Jurkat cells, NucDHS was present on HIV-IN^WT^ at 9 hpi but was evicted at 48 hpi (Fig. 5*C*). Unlike in Jurkat cells, we also observed the eviction of Nuc2 and the absence of Nuc0 sliding in primary CD4 T cells (Fig. 5 *C*–*E*). These results suggest a difference in chromatin remodeling of the viral genome between Jurkat and primary CD4 T cells. Finally, when PHA/IL2-activated primary CD4 T cells were infected with either HIV-IN^WT^ or HIV-IN^D116A^ and treated with TSA at 48 hpi, we observed transcriptional derepression of both integrated and unintegrated forms (Fig. 5*F*). This highlights the importance of chromatin in regulating transcription from unintegrated HIV DNA in primary CD4 T cells.

## Discussion

**Fig. 6.**
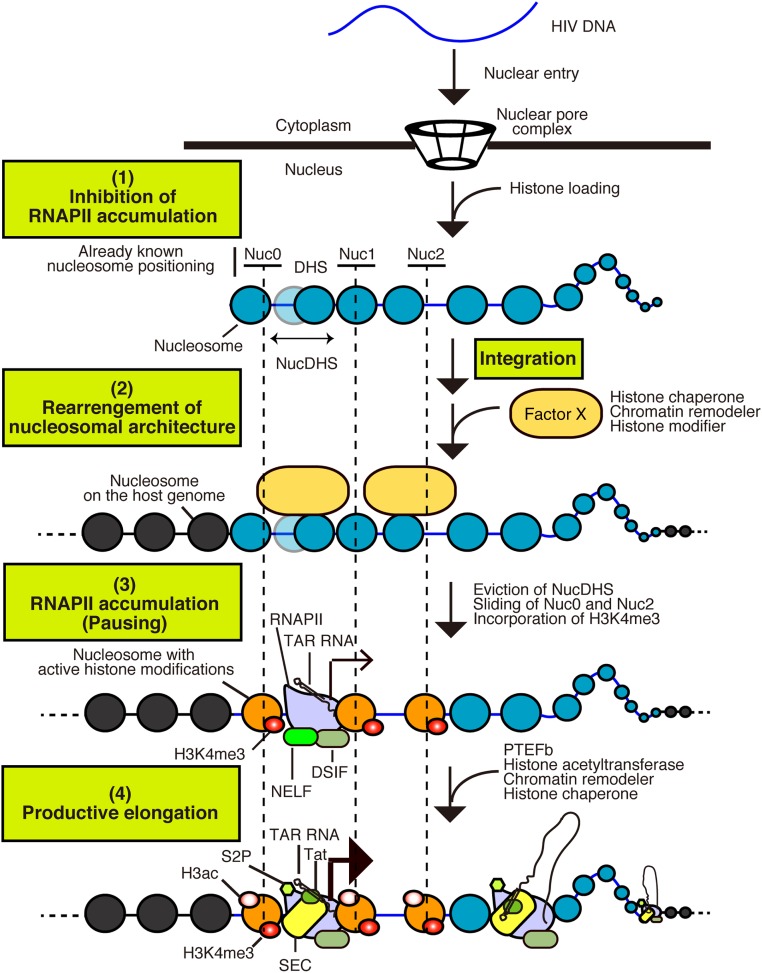
Model representing the chromatin architecture dynamics on incoming HIV DNA (1). Histones are rapidly loaded on viral DNA after its nuclear import and before its integration into the host genome. A nucleosome is formed at the DHS (NucDHS) and blocks RNAPII recruitment (2). After integration, Factor X (histone chaperones, chromatin remodelers, and histone modifiers) accumulates at the HIV LTR to rearrange the local chromatin architecture (3). RNAPII accumulates around the HIV LTR. The transition to productive transcription is inhibited through NELF-mediated pausing of RNAPII (4). Productive transcription elongation is mediated by the SEC containing PTEFb, histone acetyltransferases, chromatin remodelers, and histone chaperones.

In this study, we found that histone loading and nucleosome formation on HIV-1 DNA occur after its nuclear import and before integration in the host genome (Figs. 1, 2, and 5, and the model in Fig. 6). Comparison of our MNase-seq data for unintegrated and integrated HIV-1 DNA revealed a dynamic local chromatin architecture around the HIV-1 LTR, with eviction of a nucleosome and nucleosome sliding (Figs. 3 and 5, model in Fig. 6). Indeed, we found that in addition to the previously reported Nuc0 and Nuc1 (15, 16), an additional promoter-proximal nucleosome, NucDHS, was formed on univDNA (Figs. 3 and 5). Interestingly, both in silico nucleosome positioning and in vitro chromatin assembly on the HIV LTR revealed the presence of NucDHS (16, 33). The finding that nucleosome occupancy at the DHS region was strongly reduced in integrated DNA suggests that NucDHS is disassembled on integration (Figs. 3 and 5). Of note, since we were unable to determine nucleosome positioning along the HIV-IN^D116A^ at 48 hpi, likely due to low amounts of the 2LTR circle forms, we cannot exclude the possibility that the observed nucleosome dynamic may also occur on univDNA at a later time point. Moreover, our MNase-seq data also suggest the sliding of two nucleosomes, Nuc0 and Nuc2, localized around the LTR, on integration in Jurkat cells (Fig. 3*C*). Indeed, we found that on unintegrated vDNA, Nuc0 and Nuc2 are positioned closer to the 5′ end on the provirus. On the other hand, in PHA/IL2-activated primary CD4 T cells, we did not detect Nuc0 and Nuc2 sliding, but did observe the eviction of Nuc2 in addition to NucDHS. These results suggest a difference in the transcriptional regulation of viral LTR in these two cell models. Our ChIP and MNase-seq experiments do not discriminate between the different forms of univDNA. Further investigations are needed to analyze the specific contribution of each of the univDNA forms to the overall nucleosome positioning observed, especially given a recent study showing that the 2LTR circles are substrates for integration (34).

Nucleosome eviction and sliding are known to be involved in the regulation of various genome functions, including DNA replication, repair, and transcription (10–12); however, the cellular factors involved and how the nucleosome dynamics at the HIV LTR may affect vDNA biology, particularly viral transcription, remain unknown. Histone chaperons, ATP-dependent chromatin remodeling complexes, and histone acetyltransferases have been shown to be involved in nucleosome eviction and/or sliding (12). Interestingly, some of them, such as SWI/SNF INI1 (16, 35), SAGA (36), p300 (37, 38), and BRD4 (39, 40), have roles in vDNA integration and/or transcription. In addition, loading of the histone H2A variant H2A.Z has been associated with altered nucleosome stability (41–43). In fact, in the transcriptionally active genes, H2A.Z accumulates around TSSs that contain nucleosome-depleted regions (44–51). Additional studies are needed to identify the host factors involved in defining the chromatin architecture and the possible loading of H2A.Z at the HIV-1 LTR. Their identification will also allow definition of the timing and the effect of the observed chromatin remodeling on viral gene expression.

Promoter-proximal nucleosomes negatively regulate transcription by imposing a block on RNAPII recruitment and on the general transcription machinery (14); thus, NucDHS might have a role in preventing transcription from unintegrated HIV DNA. In line with this idea, we found that unintegrated HIV DNA was depleted in RNAPII and active histone marks, such as H3K4me3 and H3ac (Figs. 4 and 5). RNAPII, H3K4me3, and H3ac levels were increased on viral genome integration, leading to viral transcription (Figs. 4 and 5). Moreover, the finding that TSA overcomes the transcriptional repression of unintegrated HIV DNA (Figs. 4*E* and 5*F*) highlights the role of chromatin in unintegrated HIV DNA transcriptional repression.

RNAPII pausing and premature termination after synthesis of the transactivation response element (TAR; a short RNA) are hallmarks of HIV-1 gene expression (52, 53). Our findings fit with the two-step general model of the regulation of RNAPII pausing mediated by promoter-proximal nucleosomes (54). First, genes characterized by strong transcriptional pausing, such as HIV, intrinsically favor the formation of nucleosomes along the promoter to compete for RNAPII recruitment, thereby preventing aberrant transcription from paused genes (16, 33, 55, 56) (Fig. 6, *1*). Second, promoter-proximal nucleosome (NucDHS in the case of HIV) disassembly by histone chaperones, chromatin remodelers, and histone modifiers will promote gene activity by uncovering promoter motifs and favoring transcription machinery recruitment (Fig. 6, *2*). The transition to productive transcription elongation is inhibited through NELF-mediated pausing of RNAPII (17–19, 57–60) (Fig. 6, *3*). Transcription activation requires recruitment of the super elongation complex (SEC) that contains the positive transcription elongation factor b (PTEFb) to overcome NELF-mediated pausing and of chromatin remodelers to downstream nucleosomes for efficient transcription by RNAPII (52, 61–63) (Fig. 6, *4*). Identification of the host factors involved in NucDHS disassembly at HIV-1 LTR is required to understand its impact on viral gene expression.

## Materials and Methods

Detailed information on plasmids, cell culture, virus production, flow cytometry analysis, luciferase assay, quantification of viral DNA, ChIP assays, and capture MNase-seq is provided in *SI Appendix*, *Materials and Methods*.

While this manuscript was in revision, the paper by Goff’s group showing that unintegrated HIV-1 DNA is loaded with core and linker histones and is transcriptionally repressed was published (64).

## Supplementary Material

Supplementary File
