# Supplementary material for: Exploring histone loading on HIV DNA reveals a dynamic nucleosome positioning between unintegrated and integrated viral genome
Source: Proc Natl Acad Sci U S A. 2020 Mar 11;117(12):6822–30. doi: 10.1073/pnas.1913754117 (PMC7104181; doi:10.1073/pnas.1913754117)
Supplement: Supplementary File [file pnas.1913754117.sapp.pdf]

PNAS
www.pnas.org

Supplementary Information for

Exploring histone loading on unintegrated HIV DNA reveals a dynamic nucleosome positioning between unintegrated and integrated viral genome.

Shinichi Machida[a], David Depierre[b], Heng-Chang Chen[a], Suzie Thenin-Houssier[a], Gaël Petitjean[a], Cécile Doyen[a], Motoki Takaku[c], Olivier Cuvier[b] and Monsef Benkirane[a]

[a] Institut de Génétique Humaine. Université de Montpellier. Laboratoire de Virologie Moléculaire CNRS-UMR9002, 34000 Montpellier, France.
[b] Laboratoire de Biologie Moléculaire Eucaryote, Centre de Biologie Intégrative (CBI), Université de Toulouse, CNRS-UMR5099, 31000 Toulouse, France.
[c] Department of Biomedical Sciences, University of North Dakota School of Medicine, Grand Forks, ND, 58202, USA

Corresponding author: Shinichi Machida and Monsef Benkirane
Email: shinichi.machida@igh.cnrs.fr; monsef.benkirane@igh.cnrs.fr

This PDF file includes:

Supplementary text (Materials and Methods)
Figures S1 to S7
Table S1 to S4
SI References

www.pnas.org/cgi/doi/10.1073/pnas.1913754117

Materials and Methods

Plasmids

The pBR-NL4-3-Nefopt-RFP-cmv-GFP construct (further referred as HIV containing dual-color reporter), which is HIV-1 vector expressing RFP under the control of the LTR and GFP under CMV promoter, was generated on the basis of pBR-NL4-3-Nef-IRES-eGFP (1), which was gifted from F. Kirchhoff. First, DNA fragment 1 containing the cmv promoter with MluI and EcoRI restriction sites at both ends (*SI Appendix* table S1) was cloned into the MluI-NcoI site of pBR-NL4-3-Nef-IRES-eGFP with DNA fragment 2 containing spacer DNA (*SI Appendix* table S1), which contains EcoRI and NcoI restriction sites at both ends, to generate pBR-NL4-3-Nef-cmv-GFP. Second, the synthesized DNA fragment 3 containing HIV-Nefopt (*SI Appendix* table S1) was cloned into the BlpI-MluI site of pBR-NL4-3-Nef-cmv-GFP, to generate pBR-NL4-3-Nefopt-cmv-GFP. Third, the synthesized DNA fragment 4 and 5 containing RFP gene (*SI Appendix* table S1) were cloned into XhoI-MluI site of pBR-NL4-3-Nefopt-cmv-GFP, to generate pBR-NL4-3-Nefopt-RFP-cmv-GFP. pNL4-3 was obtained from the NIH AIDS Reagent Program. pHIV-Luc IN^{wt} and IN^{D116A} (Env-, HIV-1 vector that expresses firefly luciferase) were provided by S. Emiliani. The SIV3+ plasmid and pMD2-G VSV-G envelope were previously described (2).

Cell culture

Jurkat and THP-1 cells were cultured in RPMI-1640 supplemented with 10% heat-inactivated fetal bovine serum (FBS), 100 U/mL penicillin, and 100 mg/mL streptomycin. THP1 cells were differentiated with phorbol 12-myristate 13-acetate (PMA (30 ng/ml): Sigma) for overnight, and then treated with VLP containing SIVmac251 accessory protein Vpx (VLP-Vpx) for 2 hours before infection. Lenti-X 293T cells (Takara) were cultured in DMEM supplemented with 10% heat-inactivated fetal bovine serum (FBS), 2 mM ultraglutamine, 1 mM sodium pyruvate, 100 U/mL penicillin, and 100 mg/mL streptomycin. All cells were cultured at 37°C under 5% (vol/vol) CO_2.

Primary CD4 T cell isolation, culture and activation

Human primary CD4 T cells were purified with RosetteSep™ Human CD4 T Cell Enrichment Cocktail (STEMCELL Tech) from HIV-non-infected individuals, followed by isolation by Ficoll gradient method (Eurobio). To eliminate red blood cells, CD4 T cells were treated with IOTest3 lysis buffer (Beckman Coulter). After washing with PBS containing 2% heat-inactivated FBS, CD4 T cells were cultured in RPMI-1640 supplemented with 10% heat-inactivated FBS, 1X MEM Non-Essential Amino Acids Solution, 2 mM ultraglutamine, 1 mM sodium pyruvate, 100 U/mL penicillin, and 100 mg/mL streptomycin. After 3 hours at 37°C under 5% (vol/vol) CO_2, CD4 T cells were stimulated with 0.5 µg/ml phytohaemagglutinin (PHA, Sigma) and 50 U/ml interleukin 2 (IL-2, Roch) for 3 days.

Virus production

Viral particles and VLP were produced from Lenti-X 293T cells by the standard phosphate calcium transfection method. Briefly, 293T cells were transfected with 8 µg viral vectors (pBR-NL4-3-Nefopt-RFP-cmv-GFP, pNL4-3 IN^{wt} or IN^{D116A}, or pHIV-Luc IN^{wt} or IN^{D116A}) and 2 µg of pMD2-G VSV-G-encoding plasmid, medium was replaced 16 hours later. After 48 hours, viruses were harvested, filtered (0.45 µm), and concentrated by the ultracentrifugation. Viruses were treated with 0.1 U/µl DNase (TURBO DNase, Thermo Fisher Scientific) at 37 °C for 1 hour. The p24 concentration of viral stocks was measured by ELISA (Zeptometrix), and multiplicity of infection was calculated by titration in Jurkat cells. For virus-like particles (VLP) containing $SIVmac_{251}$ accessory protein Vpx ($VLP\text{-}Vpx_{mac251}$), 293T cells were transfected with 8 µg SIV3+ plasmid and 2 µg pMD2-G VSV-G-encoding plasmid, and medium was replaced 16 hours later. After 48 hours, $VLP\text{-}Vpx_{mac251}$ were harvested, and filtered (0.45 µm).

Flow Cytometry Analysis

Jurkat cells were infected with VSV-G pseudotyped HIV-1 containing the dual-color reporter at the multiplicity of infection of 0.2 (calculated by GFP expression). After 3 hours, the cell culture medium was changed. Cells were fixed with 1% paraformaldehyde at 9, 24 and 48 hours post-

infection (hpi), and subjected to flow cytometry analysis using a MACSQuant Analyzer (Miltenyi Biotec). Data were analyzed using FlowJo software.

Luciferase assay
Jurkat cells were infected with HIV-Luc IN^{wt} or IN^{D116A} at the multiplicity of infection of 0.5. After 3 hours, the cell culture medium was changed. Subsequently, TSA (sigma: 0.5 μM) was added in the medium at 32 hpi, and cells were lysed in passive lysis buffer (Promega) at 48 hpi. Insoluble proteins were pelleted by centrifugation at 16,300 g, and supernatant was subjected to Luciferase assay system (Promega). The protein concentration was measured by Pierce BCA protein assay kit (Thermo Fisher Scientific). Luminescence was measured by TriStar LB 941 (Berthold technologies), and normalized by the protein concentration.
For experiments using primary CD4 T cells, CD4 T cells activated for 3 days were infected with HIV-Luc IN^{wt} or IN^{D116A} at the multiplicity of infection of 0.5. After 3 hours, the cell culture medium was changed. TSA (0, 0.125, 0.5, 2, 8 μM) was added in the medium at 32 hpi, and cells were lysed in passive lysis buffer at 48 hpi.

Quantification of viral DNA
Jurkat cells were infected with VSV-G pseudotyped HIV-1 containing the dual-color reporter at the multiplicity of infection of 0.2 (calculated by GFP expression) in the presence or absence of 1 μM raltegravir (Selleckchem). After 3 hours, the cell culture medium was changed. Cells were collected at 3, 6, 9, 12, 24, and 48 hpi, DNA was extracted using DNeasy Blood and Tissue Kit (Qiagen) and subjected to qPCR. For quantification of total HIV DNA, 25 ng DNA was used in each reaction (3,4). For quantification of 2LTR circle, 100 ng DNA was used in each reaction. Integrated HIV DNA was quantified by Alu-PCR method as described previously (4), using 100 ng extracted DNA. Total HIV DNA, 2LTR circle, and integrated HIV DNA were normalized by β-globin. Primer sequences were listed in *SI Appendix* table S2. For experiments using PF74, differentiated THP-1 cells were treated with VLP-Vpx, and infected with VSV-G pseudotyped HIV-1 containing the dual-color reporter in the presence or absence of 2 μM PF74 (Sigma). The cell culture medium was changed 3 hours later, and cells were collected at 24 hpi. DNA was then extracted using the DNeasy Blood and Tissue Kit (Qiagen) and subjected to qPCR. All drugs were added in the medium for the entire duration of infection.

ChIP assay
Jurkat cells were infected with VSV-G pseudotyped HIV-1 containing the dual-color reporter at the multiplicity of infection of 0.2 (calculated by GFP expression) in the presence or absence of 1 μM raltegravir (Selleckchem) or nevirapine (Sigma). For experiments using primary CD4 Tcells, CD4 T cells activated for 3 days were infected with VSV-G pseudotyped NL4-3 IN^{wt} or IN^{D116A} at the multiplicity of infection of 0.4. After 3 hours, the cell culture medium was changed. Cells were collected at 9 and 48 hpi, and fixed with 1% formaldehyde for 10 min at room temperature. Chromatin immunoprecipitation reactions were performed using SimpleChIP Plus Enzymatic Chromatin IP Kit (Cell Signaling Technology) according to the protocol provided by the manufacturer. 13-16 μg of chromatin was digested with MNase (Roche) and reacted with 4 μg of respective antibodies. qPCRs were performed using specific primers (*SI Appendix* table S3)
For experiments using PF74, differentiated THP-1 cells were treated with VLP-Vpx and infected with VSV-G pseudotyped HIV-1 containing the dual-color reporter in the absence or presence of 2 μM PF74 (Sigma). The cell culture medium was changed 3 hours later. Cells were collected at 24 hpi, and fixed with 1% formaldehyde for 10 min at room temperature. Chromatin immunoprecipitation reactions were performed using a ChIP-IT PBMC Kit (Active Motif) according to the protocol provided by the manufacturer, except for the nucleus preparation step. Fixed cells were directly lysed by ChIP buffer (Active Motif), and sonicated with Bioruptor Pico (Diagenode). 30-40 μg of sonicated chromatin were reacted with 4 μg of anti-Histone H3 antibody. qPCRs were performed using specific primers (*SI Appendix* table S3).
The following antibodies were used for ChIP: anti-histone H3 (Abcam, ab1791), anti-histone H2B (Abcam, ab1790), anti-histone H3ac (Millipore, 06-599), anti-histone H3K4me3 (Abcam, ab8580), and anti-RNAPII (Santa Cruz Biotechnology, F12 sc-55492) antibody.

Capture MNase-seq
Jurkat cells ($1x10^7$) were infected with VSV-G pseudotyped NL4-3 IN^{wt} or IN^{D116A} at the multiplicity of infection of 0.2. For experiments using primary CD4 Tcells, CD4 T cells ($0.5x10^7$) activated for 3 days were infected with VSV-G pseudotyped NL4-3 IN^{wt} or IN^{D116A} at the multiplicity of infection of 0.2. After 3 hours, the cell culture medium was changed. Cells were collected at 9 and 48 hpi, and native mononucleosomes were prepared as previously described (5). Briefly, $2x10^6$ cells were treated with hypotonic buffer (10 mM Tris-HCl pH 7.5, 10 mM NaCl, 3 mM $MgCl_2$, 0.5% Triton X-100) for 15 min on ice, followed by centrifugation. Pellets were washed with 300 µl of MNase digestion buffer (10 mM Tris-HCl pH 7.5, 15 mM NaCl, 60 mM KCl, 3 mM $CaCl_2$). The nuclei were resuspended with 100 µl of MNase digestion buffer, and treated with 2.1 U of MNase at 37 °C for 4 min. Reactions were terminated by adding 150 µl of stop buffer (20 mM EDTA, 20 mM EGTA, 0.4% SDS, and 0.5 mg/ml proteinase K), and incubate at 55 °C for 6 hours or overnight. DNA was then extracted by phenol–chloroform extraction, followed by ethanol precipitation. Mononucleosome-sized DNA was separated by 2% agarose gel electrophoresis, and purified using QIAGEN gel extraction kit. To obtain sonicated fragments, DNA was extracted from nuclei washed with MNase digestion buffer using DNeasy Blood and Tissue Kit (Qiagen), and sonicated with Bioruptor Pico (Diagenode) to produce an average DNA fragment size of 100–300 bp.
Libraries were prepared using NEXTflex Rapid DNA-Seq kit2.0 (BIOO SCIENTIFIC) using 100 ng input DNA according to the protocol provided by the manufacturer. To capture library DNAs containing HIV-1 sequence, the custom-designed capture bait system, consisting of 804 biotinylated RNAs targeting entire pNL4-3 and pHRET (2) except for the sequence of the vector, was designed according to the manufacturer's instructions (Agilent Technologies). Library DNAs (600-800 ng) were concentrated using a vacuum concentrator at below 45°C, and reconstitute with H_2O to obtain 3.4 µl total volume. Library DNAs were then hybridized with capture bait RNAs according to the protocol provided by the manufacturer (SureSelect XT Target Enrichment, Agilent Technologies). Briefly, SureSelect Block Mix were added to library DNAs, and were heated at 95°C for 5 min, followed by an incubation at 65°C for more than 5 min. Subsequently, Capture Library Hybridization Mix, containing Custom Design SureSelect XT Capture Libraries and SureSelect RNase Block, was mixed with library DNAs, and was incubated at 65°C for 24 hours. To capture the hybridized DNA, MyOne Streptavidin T1 Dynabeads (Life Technologies) were mixed with hybridized library DNAs. After incubation at 25°C for 30 min with mixing at 1400 rpm, beads were washed with SureSelect Wash Buffer according to SureSelect XT Target Enrichment protocol. After washing, 30 µl H_2O was added to beads. Post-capture PCR was carried out using DNAs bound to beads via biotinylated-RNAs (16 cycles using NEXTflex primer mix2 (BIOO SCIENTIFIC)). Captured MNase-seq libraries were paired-end sequenced on Illumina HiSeq 2500.

Processing and analysis of MNase-seq data
Paired-end Mnase-seq reads were trimmed of adapter sequences (cutadapt 1.8.3) using Trim Galiore tool (Trim Galore 0.5.0) together with a quality control step (FastQC v0.11.7). Clean reads were aligned on viral HIV DNA pNL4-3 sequence (GenBank: AF324493.2) using Burrows-Wheeler Aligner (BWA 0.7.15, http://bio-bwa.sourceforge.net/). The aligned reads were filtered and sorted using Samtools 1.8. Duplicated reads (e.g. due to PCR) were removed using picard tools (picard-2.18.2). When visualizing reads onto LTRs, multiple alignment option was chosen to keep reads aligning onto such repeated sequences. In this instance, multiple reads were normalized to the number of copies of viral genome detected in cells. For specific detection of nucleosome profiles, alignment were performed using Deeptools bamCoverage (deeptools-3.0.2, python-3.4.3) by filtering fragment of sizes between 130 bp and 190 bp with the Mnase 'center' option to get the site of the nucleosome center. For sonicated samples, a classic bamCoverage was applied without any filter or resize of fragment size. All analysis and visualization were done with R 3.4.2 version. Profiles were plotted using SeqPlots R package from Bioconductor (SeqPlots 1.16.0).
A differential score was calculated as

$$Zscore = \frac{(KD - WT)}{\sqrt{(KD + WT)/_2}}$$

(i.e. evaluating differences weighted by the mean signal) to compare nucleosome-centered signals. Differences between conditions (2x2 replicates) were validated statistically using the NormR R package designed for differential analysis of sequencing data. The diffR function was applied to compare samples coverage with the following configuration: ”countConfiguration<-countConfigPairedEnd(binsize = 50 ,mapq = 0, shift = 0, midpoint=T, tlenFilter = c(130, 200))” allowing us to detect systematically differences at the nucleosome scale. Differences detected were filtered with a FDR < 1e-3. Differential heatmaps were then filtered to represent only loci where decreases or increases were significantly validated with a FDR < 1e-3.

Data and software availability
The accession number for the data reported in this paper is GEO (GSE139557(GSE135551 and GSE139556)).
Software and Algorithms were listed in *SI Appendix* table S4.

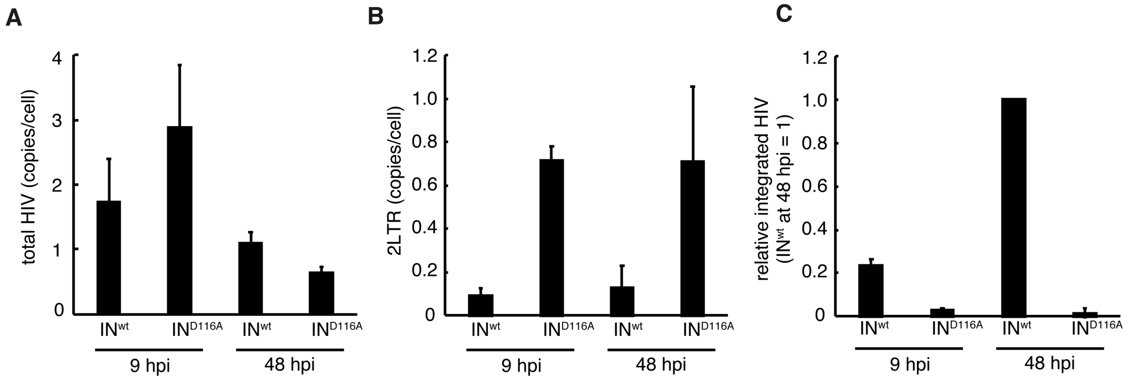

Fig. S1. Quantification of HIV-1 DNA in Jurkat cells infected with HIV-IN^{wt} or IN^{D116A}. Jurkat cells infected with VSV-G pseudotyped NL4-3 IN^{wt} or IN^{D116A} were treated with hypotonic buffer (10 mM Tris-HCl pH 7.5, 10 mM NaCl, 3 mM MgCl2, 0.5% Triton X-100). After washing with MNase digestion buffer (10 mM Tris-HCl pH 7.5, 15 mM NaCl, 60 mM KCl, 3 mM CaCl2), DNA was extracted. Total HIV DNA, 2LTR circle, and integrated HIV DNA were quantified by qPCR. Total HIV (A) and 2LTR (B) were shown as copy numbers per cell. Integrated HIV DNA (C) were represented as the relative quantification to the value of IN^{wt} at 48 hpi. The average values of three independent experiments are plotted with SD values.

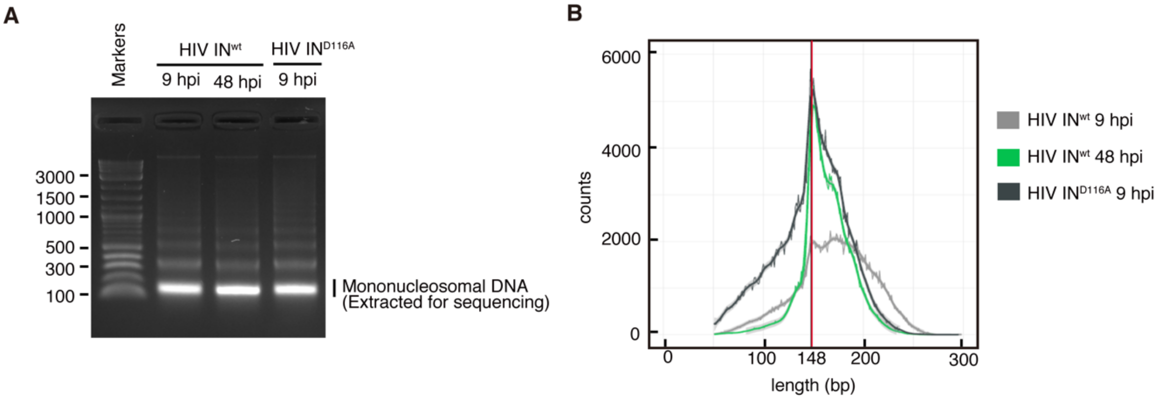

Fig. S2. Fragment length distribution of the captured MNase-seq libraries (HIV-infected Jurkat cells).

(A) Chromatin treated with MNase was deproteinized and analyzed by 1.5 % agarose gel electrophoresis stained with SYBR Safe.

(B) Fragment length distribution of the reads aligned on HIV DNA. The theoretical nucleosomal DNA length (148 bp) is indicated with a red line.

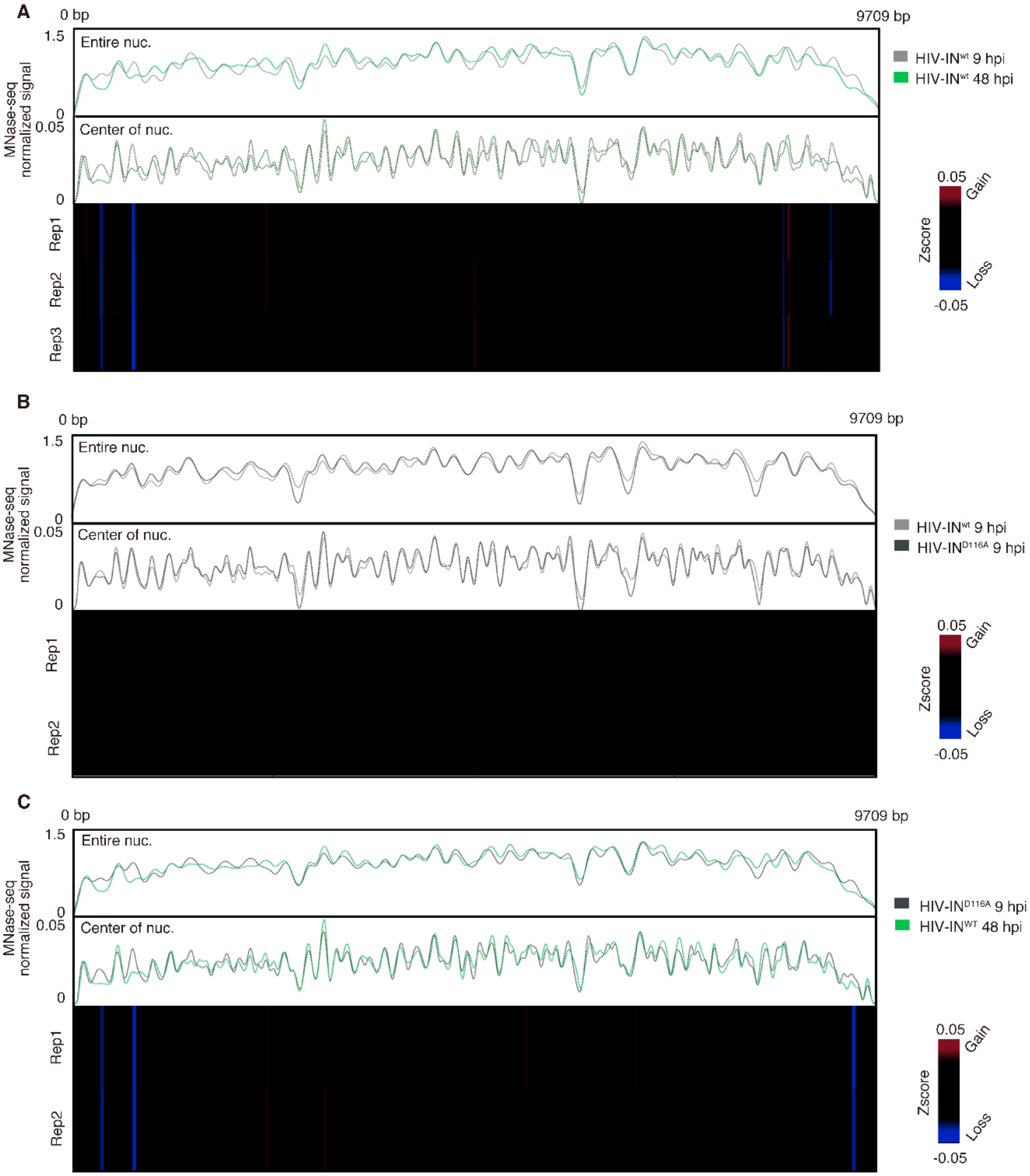

Fig. S3. MNase-seq profile over the entire HIV-1 DNA (HIV infected Jurkat cells).
(A) MNase-seq profile over the entire HIV-1 DNA comparing HIV-IN^{wt} at 9 and 48 hpi. The first panel corresponds to coverage with fragment extended at 148 bp to fit theoretical nucleosome size. The second panel is coverage corresponding to the center of nucleosome to sharply confirm their position (see *SI Appendix Materials and Methods*). The third panel corresponds to the differential heatmap showing variation in nucleosome coverage over the entire HIV-1 DNA, with blue as a loss of signal and red as a gain of signal from HIV-IN^{wt} at 9 hpi to HIV-IN^{wt} at 48 hpi. Differential heatmap are filtered to show only significantly validated differential locus with a FDR < 1e-3 (see *SI Appendix Materials and Methods*).
(B) MNase-seq profile over the entire HIV-1 DNA comparing HIV-IN^{wt} to HIV-IN^{D116A} at 9 hpi. description of the panels is similar to those in (A).

(C) MNase-seq profile over the entire HIV-1 DNA comparing HIV-IN^{D116A} at 9 hpi to HIV-IN^{wt} at 48 hpi. descriptions of the panels is similar to those in (A).

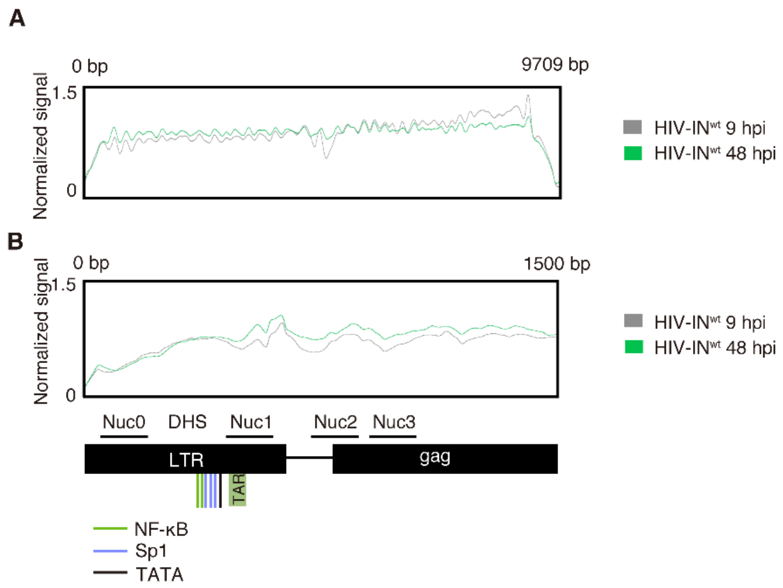

Fig. S4. Genome coverage of sonicated HIV DNA fragments (HIV infected Jurkat cells).
(A) Genome coverage comparing sonicated samples HIV-IN^{WT} at 9 and 48 hpi over the entire HIV-1 DNA.
(B) Genome coverage comparing sonicated samples HIV-IN^{WT} at 9 and 48 hpi over first 1500bp of the HIV-1 DNA. The genome annotation was represented with binding sites for NF-κB and SP1, TATA, and TAR. Already described nucleosome positions in the integrated vDNA are also represented in the genome annotation.

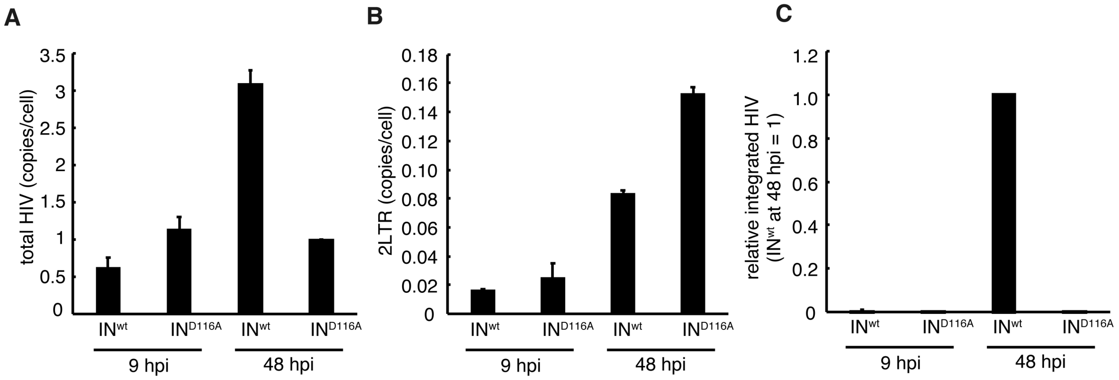

Fig. S5. Quantification of HIV-1 DNA (HIV infected CD4 T cells).
Primary CD4 T cells infected with VSV-G pseudotyped NL4-3 IN^{wt} or IN^{D116A} were treated with hypotonic buffer (10 mM Tris-HCl pH 7.5, 10 mM NaCl, 3 mM MgCl2, 0.5% Triton X-100). After washing with MNase digestion buffer (10 mM Tris-HCl pH 7.5, 15 mM NaCl, 60 mM KCl, 3 mM CaCl2), DNA was extracted. Total HIV DNA, 2LTR circle, and integrated HIV DNA were quantified by qPCR. Total HIV (A) and 2LTR (B) were shown as copy numbers per cell. Integrated HIV DNA (C) were represented as the relative quantification to the value of IN^{wt} at 48 hpi. The average values of two independent experiments are plotted with SD values.

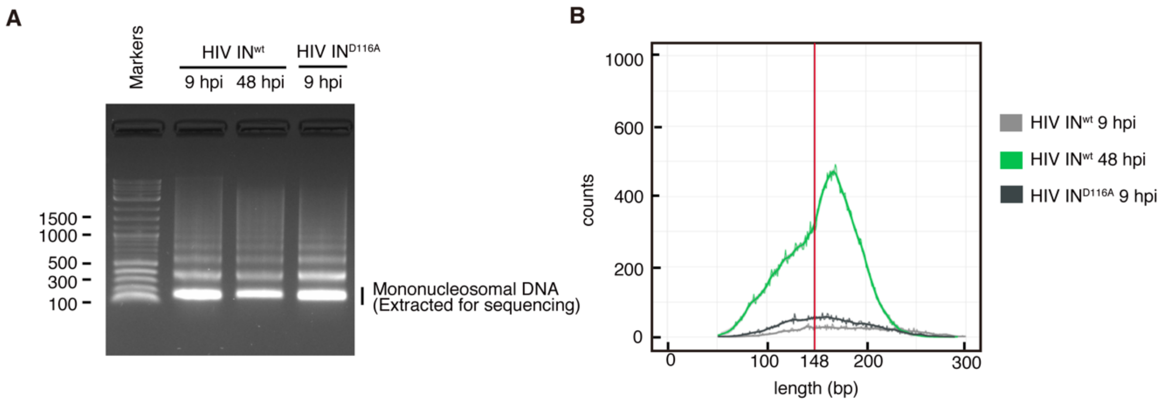

Fig. S6. Fragment length distribution of the captured MNase-seq libraries (HIV infected CD4 T cells).

(A) Chromatin treated with MNase was deproteinized and analyzed by 1.5 % agarose gel electrophoresis stained with SYBR Safe.

(B) Fragment length distribution of the reads aligned on HIV DNA. The theoretical nucleosomal DNA length (148 bp) is indicated with a red line.

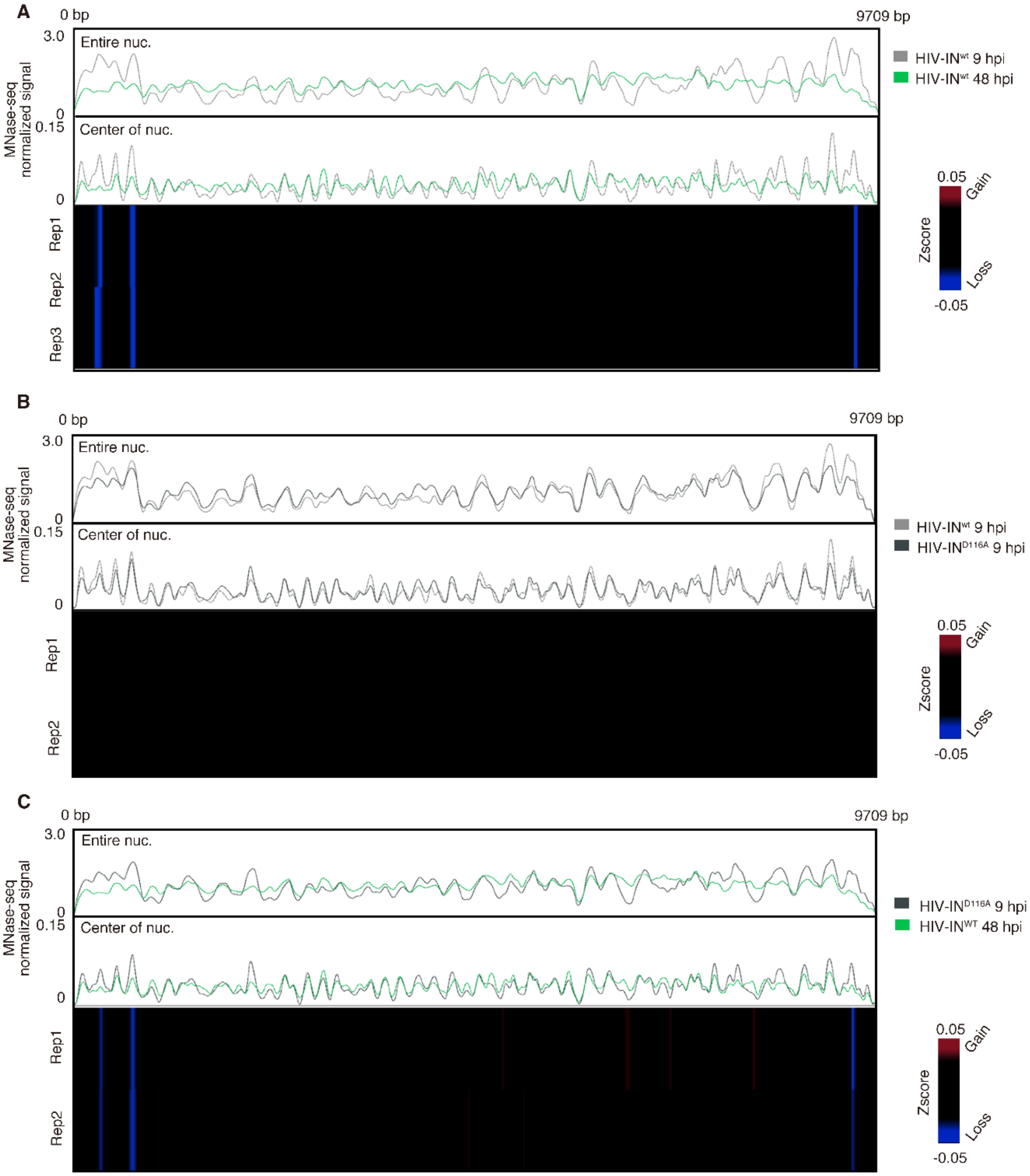

Fig. S7. MNase-seq profile over the entire HIV-1 DNA (HIV infected CD4 T cells).
(A) MNase-seq profile over the entire HIV-1 DNA comparing HIV-IN^{wt} at 9 and 48 hpi. The first panel corresponds to coverage with fragment extended at 148 bp to fit theoretical nucleosome size. The second panel is coverage corresponding to the center of nucleosome to sharply confirm their position (see *SI Appendix Materials and Methods*). The third panel corresponds to the differential heatmap showing variation in nucleosome coverage over the entire HIV-1 DNA, with blue as a loss of signal and red as a gain of signal from HIV-IN^{wt} at 9 hpi to HIV-IN^{wt} at 48 hpi. Differential heatmap are filtered to show only significantly validated differential locus with a FDR < 1e-3 (see *SI Appendix Materials and Methods*).
(B) MNase-seq profile over the entire HIV-1 DNA comparing HIV-IN^{wt} to HIV-IN^{D116A} at 9 hpi. description of the panels is similar to those in (A).

(C) MNase-seq profile over the entire HIV-1 DNA comparing HIV-IN^{D116A} at 9 hpi to HIV-IN^{wt} at 48 hpi. descriptions of the panels is similar to those in (A).

Table S1. DNA fragments used in the plasmid construction.

Name	Sequence (5’-3’)
DNA fragment 1 containing the cmv promoter	ACGCGTATTGCATTACTAGTTATTAATAGTAATCAATTACGGGGTCATTAGTTCA<br>TAGCCCATATATGGAGTTCCGCGTTACATAACTTACGGTAAATGGCCCGCCTGG<br>CTGACCGCCCAACGACCCCCGCCCATTGACGTCAATAATGACGTATGTTCCCA<br>TAGTAACGCCAATAGGGACTTTCCATTGACGTCAATGGGTGGAGTATTTACGGT<br>AAACTGCCCACTTGGCAGTACATCAAGTGTATCATATGCCAAGTCCGCCCCCTA<br>TTGACGTCAATGACGGTAAATGGCCCGCCTGGCATTATGCCCAGTACATGACC<br>TTACGGGACTTTCCTACTTGGCAGTACATCTACGTATTAGTCATCGCTATTACCA<br>TGGTGATGCGGTTTTGGCAGTACACCAATGGGCGTGGATAGCGGTTTGACTCA<br>CGGGGATTTCCAAGTCTCCACCCCATTGACGTCAATGGGAGTTTGTTTTGGCAC<br>CAAAATCAACGGGACTTTCCAAAATGTCGTAATAACCCCGCCCCGTTGACGCAA<br>ATGGGCGGTAGGCGTGTACGGTGGGAGGTCTATATAAGCAGAGCTCGTTTAGT<br>GAACCGTCAGAATTGTTTTTATTTTTAATTTTCTTTCAAATACTTCCATCGAATTC
DNA fragment 2 containing spacer DNA	AATTCGTACGCCTTACGCCGCCACCATG
DNA fragment 3 containing HIV-Nefopt	GCTGAGCCAGCAGCAGATGGGGTGGGAGCAGTATCTCGAGACCTAGAAAAAC<br>ATGGAGCAATCACAAGTAGCAATACAGCAGCTAACAATGCTGCTTGTGCCTGG<br>CTAGAAGCACAAGAGGAGGAAGAGGTGGGTTTTCCAGTCACACCTCAGGTACC<br>TTTAAGACCAATGACTTACAAGGCAGCTGTAGATCTGAGCCATTTCCTGAAGGA<br>GAAGGGAGGCCTTGAGGGCCTGATTCACAGCCAGCGGCGCCAAGACATCCTT<br>GACTTGTGGATTTACCATACACAGGGGTACTTCCCCGACTGGCAGAACTATACA<br>CCAGGACCCGGAGTGAGATACCCCCTGACCTTCGGATGGTGTTACAAACTGGT<br>TCCAGTGGAGCCTGATAAGGTCGAAGAGGCAAACAAAGGCGAGAATACATCTT<br>TGCTGCATCCTGTGTCACTGCACGGGATGGACGACCCTGAGCGGGAGGTGCT<br>TGAGTGGAGGTTCGACTCTCGACTGGCCTTTCACCACGTAGCAAGGGAGCTGC<br>ACCCTGAGTATTTTAAAAATTGTTGAACGCGT
DNA fragment 4 containing RFP gene	CTCGAGACCTAGAAAAACATGGAGCAATCACAAGTAGCAATACAGCAGCTAACA<br>ATGCTGCTTGTGCCTGGCTAGAAGCACAAGAGGAGGAAGAGGTGGGTTTTCCA<br>GTCACACCTCAGGTACCTTTAAGACCAATGACTTACAAGGCAGCTGTAGATCTG<br>AGCCATTTCCTGAAGGAGAAGGGAGGCCTTGAGGGCCTGATTCACAGCCAGC<br>GGCGCCAAGACATCCTTGACTTGTGGATTTACCATACACAGGGGTACTTCCCC<br>GACTGGCAGAACTATACACCAGGACCCGGAGTGAGATACCCCCTGACCTTCGG<br>ATGGTGTTACAAACTGGTTCCAGTGGAGCCTGATAAGGTCGAAGAGGCAAACA<br>AAGGCGAGAATACATCTTTGCTGCATCCTGTGTCACTGCACGGGATGGACGAC<br>CCTGAGCGGGAGGTGCTTGAGTGGAGGTTCGACTCTCGACTGGCCTTTCACCA<br>CGTAGCAAGGGAGCTGCACCCTGAGTATTTTAAAAATTGTAGCGAGCTGATCAA<br>GGAGAACATGC
DNA fragment 5 containing RFP gene	ACATGCACATGAAGCTGTACATGGAGGGCACCGTGAACAACCACCACTTCAAG<br>TGCACATCCGAGGGCGAAGGCAAGCCCTACGAGGGCACCCAGACCATGAAGA<br>TCAAGGTGGTCGAGGGCGGCCCTCTCCCCTTCGCCTTCGACATCCTGGCTACC<br>AGCTTCATGTACGGCAGCAAAGCCTTCATCAACCACACCCAGGGCATCCCCGA<br>CTTCTTTAAGCAGTCCTTCCCTGAGGGCTTCACATGGGAGAGAATCACCACATA<br>CGAAGACGGGGGCGTGCTGACCGCTACCCAGGACACCAGCTTCCAGAACGGC<br>TGCATCATCTACAACGTCAAGATCAACGGGGTGAACTTCCCATCCAACGGCCCT<br>GTGATGCAGAAGAAAACACGCGGCTGGGAGGCCAACACCGAGATGCTGTACC<br>CCGCTGACGGCGGCCTGAGAGGCCACAGCCAGATGGCCCTGAAGCTCGTGGG<br>CGGGGGCTACCTGCACTGCTCCTTCAAGACCACATACAGATCCAAGAAACCCG<br>CTAAGAACCTCAAGATGCCCGGCTTCCACTTCGTGGACCACAGACTGGAAAGA<br>ATCAAGGAGGCCGACAAAGAGACCTACGTCGAGCAGCACGAGATGGCTGTGG<br>CCAAGTACTGCGACCTCCCTAGCAAACTGGGGCACAGATAACGCGT

Table S2. Primers used in quantification of the HIV DNA.

Name	Sequence (5'-3')	Target	Reference
MH531	TGTGTGCCCGTCTGTTGTGT	Total HIV-1 DNA	(3,4)
MH532	GAGTCCTGCGTCGAGAGAGC	Total HIV-1 DNA	(3,4)
2LTR-F	GTGCCCGTCTGTTGTGTGACT	2LTR circle	(4)
2LTR-R	TGCTTATATGCAGCATCTGAGG	2LTR circle	This paper
2LTR-R2	ACTGGTACTAGCTTGTAGCAC CATCCA	2LTR circle (Fig. S1 and S5)	(4)
L-M667	ATGCCACGTAAGCGAAACTCT GGCTAACTAGGGAACCCACTG	Integrated HIV (Alu-PCR (1st round PCR))	(4)
Alu 1	TCCCAGCTACTGGGGAGGCTG AGG	Integrated HIV (Alu-PCR (1st round PCR))	(4)
Alu 2	GCCTCCCAAAGTGCTGGGATT ACAG	Integrated HIV (Alu-PCR (1st round PCR))	(4)
Lambda T	ATGCCACGTAAGCGAAACT	Integrated HIV (Alu-PCR (2nd round PCR))	(4)
AA55M	GCTAGAGATTTTCCACACTGAC TAA	Integrated HIV (Alu-PCR (2nd round PCR))	(4)
β-globin-F	CCCTTGGACCCAGAGGTTCT	β-globin	(6)
β-globin-R	CGAGCACTTTCTTGCCATGA	β-globin	(6)

Table S3. Primers for ChIP qPCR.

Name	Sequence (5’-3’)	Reference
DHS-F	TCCGGAGTACTTCAAGAACTGC	This paper
DHS-R	TGCTTATATGCAGCATCTGAGG	This paper
Nuc1-F	GAGTGCTCAAAGTAGTGTGTGC	This paper
Nuc1-R	TCTCCTCTGGCTTTACTTTCGC	This paper
Nuc2-F	GCGACTGGTGAGTACGCCAA	(7)
Nuc2-R	CCCCTGGCCTTAACCGAATTT	(7)
Vif-F	GAGAAAGAGACTGGCATTTGGG	This paper
Vif-R	GATGAATTAGTTGGTCTGCTAGGTC	This paper
RFP-F	TCCCCGACTTCTTTAAGCAGTC	This paper
RFP-R	AGTTCACCCCGTTGATCTTGAC	This paper
GAPDH-Pro.-F	TACTAGCGGTTTTACGGGCG	Sigma
GAPDH-Pro.-R	TCGAACAGGAGGAGCAGAGAGCGA	Sigma
GAPDH-Cod.-F	GGCTCCCACCTTTCTCATCC	Sigma
GAPDH-Cod.-R	GGCCATCCACAGTCTTCTGG	Sigma
B13-F	GAAACCACCCACGTGTTCTTTC	This paper
B13-R	TGGATCCCTGATCACCTCCAAG	This paper

Table S4. Software and algorithms for data processing

Name	Reference
Trim Galore 0.5.0	(8)
Cutadapt 1.8.3	(9)
FastQC v0.11.7	(10)
Burrows-Wheeler Alignement BWA 0.7.15	(11)
Samtools 1.8	(12)
picard-2.18.2	(13)
deeptools-3.0.2, python-3.4.3	(14)
R Project for Statistical analysis R 3.4.2	https://www.r-project.org/
Bioconductor	https://www.bioconductor.org/
SeqPlots 1.16.0	(15)
NormR package	(16)

SI References

1. J. Munch, et al., Nef-mediated enhancement of virion infectivity and stimulation of viral replication are fundamental properties of primate lentiviruses. *J. Virol.* **81**, 13852–13864 (2007)
2. B. Descours, et al., CD32a is a marker of a CD4 T-cell HIV reservoir harbouring replication-competent proviruses. *Nature* **543,** 564–567 (2017)
3. S.L. Butler, M.S. Hansen, F.D. Bushman, A quantitative assay for HIV DNA integration in vivo. *Nat. Med.* **7**, 631-634 (2001)
4. A. Brussel, P. Sonigo, Analysis of early human immunodeficiency virus type 1 DNA synthesis by use of a new sensitive assay for quantifying integrated provirus. *J. Virol.* **77**, 10119–10124 (2003)
5. K. Cui, K. Zhao, Genome-wide approaches to determining nucleosome occupancy in metazoans using MNase-seq. Methods Mol. Biol. **833**, 413–419 (2011)
6. U. O'Doherty, W.J. Swiggard, M.H. Malim, Human immunodeficiency virus type 1 spinoculation enhances infection through virus binding. *J. Virol.* **74**, 10074–10080 (2000)
7. C.F. Kessing, et al., In vivo suppression of HIV rebound by didehydro-cortistatin A, a "'block-and-lock'" strategy for HIV-1 treatment. *Cell Rep.* **21**, 600–611 (2017)
8. F. Krueger, A wrapper tool around Cutadapt and FastQC to consistently apply quality and adapter trimming to FastQ files, with some extra functionality for MspI-digested RRBS-type (Reduced Representation Bisufite-Seq) libraries. Available online at: https://www.bioinformatics.babraham.ac.uk/projects/trim_galore/ (2012)
9. M. Martin, Cutadapt removes adapter sequences from high-throughput sequencing reads. EMBnet. Journal **17**, 10–12 (2011)
10. S. Andrews, FastQC: a quality control tool for high throughput sequence data. Available online at: http://www.bioinformatics.babraham.ac.uk/projects/fastqc (2010)
11. H. Li, R. Durbin, Fast and accurate short read alignment with Burrows–Wheeler transform. *Bioinformatics* **25**, 1754–1760 (2009)
12. H. Li, et al., The sequence alignment/map format and SAMtools. *Bioinformatics* **25**, 2078–2079 (2009)
13. Broad Institute, Picard Toolkit : A set of command line tools (in Java) for manipulating high-throughput sequencing (HTS) data and formats such as SAM/BAM/CRAM and VCF. Available online at: http://broadinstitute.github.io/picard/ (2019)
14. F. Ramírez, F. Dündar, S. Diehl, B.A. Grüning, T. Manke, deepTools: a flexible platform for exploring deep-sequencing data. *Nucleic Acids Res.* **42**, W187–W191 (2014)
15. P. Stempor, J. Ahringer, SeqPlots - Interactive software for exploratory data analyses, pattern discovery and visualization in genomics. Wellcome Open Research 1. R package version 1.22.2, https://wellcomeopenresearch.org/articles/1-14. (2014)
16. J. Helmuth, H. Chung, normr: Normalization and difference calling in ChIP-seq data. R package version 1.10.0. Available online at: https://github.com/your-highness/normR. (2019)
